# Remotely Monitored Patients' Experiences of the Interpersonal Patient–Nurse Relationship: A Scoping Review

**DOI:** 10.1111/scs.70166

**Published:** 2025-12-15

**Authors:** Anna Granath, Stine Eileen Torp Løkkeberg, Wivica Kauppi, Fredrik Andersen, Leif Sandsjö, Erik Eriksson

**Affiliations:** ^1^ Faculty of Caring Science, Work Life and Social Welfare, Dept of Caring Science University of Borås Borås Sweden; ^2^ Faculty of Health, Welfare and Organisation, Dept of Welfare, Management and Organisation Østfold University College Halden Norway; ^3^ Faculty of Child Protection, Social Work and Social Education, Dept of Social Work and Guidance University of Inland Lillehammer Norway; ^4^ PreHospen‐ Centre for Prehospital Research Faculty of Caring Science, Work Life and Social Welfare, University of Borås Borås Sweden; ^5^ Faculty of Caring Science, Work Life and Social Welfare, Dept of Work Life and Social Welfare University of Borås Borås Sweden; ^6^ Industrial and Materials Science, Division of Design & Human Factors Chalmers University of Technology Gothenburg Sweden

**Keywords:** caring, eHealth, health data, interpersonal relationships, literature review, nurse–patient relationship, patient experience, person‐centred care, remote monitoring, telehealth

## Abstract

**Objective:**

To explore what has been published in peer‐reviewed journals on patients' experiences of the interpersonal relationship between the patient and the nurse when the patient's health data are remotely monitored in an out‐of‐clinic setting.

**Introduction:**

Interpersonal relationships are considered a cornerstone in person‐centred care and nursing. These relationships can be influenced by context and environment. Remote patient monitoring is increasing within healthcare, with the potential to impact on the relationship between the patient and the nurse. So far, there has been limited knowledge on a general basis of how remotely monitored patients experience this relationship.

**Inclusion Criteria:**

Original peer‐reviewed studies in English, published year 2014–2024. Patients 18 years and over in an out‐of‐clinic setting, having their health data collected through remote patient monitoring by nurses exclusively or as part of a multiprofessional team. Patients' experiences/attitudes/perspectives/perceptions of the patient–nurse relationship.

**Methods:**

The research method was based on the Joanna Briggs Institute's method for scoping reviews and the PRISMA checklist for scoping reviews was used when reporting the review. In total, four databases were used in the literature search. Thematic analysis was used for analysing the results.

**Results:**

Out of 9001 articles, 31 studies were included in the review. Thematic analysis resulted in three clusters covering relational aspects and emotional responses of the patient–nurse relationship during remote monitoring. These clusters were *Relational aspects of patient*–*nurse communication and interaction*, *Emotional aspects of the patient*–*nurse relationship* and *Patient participation*.

**Conclusions:**

Remote patient monitoring is primarily a human‐to‐human activity. In general, but not solely, patients perceive positive relational experiences with nurses during remote monitoring. Remote patient monitoring can and should be delivered with a person‐centred and ethically aware approach. Thus, the acts and efforts of the monitoring nurse play a central role in providing a positive relationship‐based experience during remote patient monitoring.

## 1 Introduction

Interpersonal relationships are an important part of everyday life, including within the healthcare context. The patient–nurse relationship is a central element of fundamental care [1]. Fundamental care is related to person‐centred care (PCC), with the patient at the centre of the care and the caregiver maintaining a holistic approach [2]. The foundation of PCC comprises patient involvement, clinician‐patient relationship and context [3], where the uniqueness and involvement of the individual is encouraged and a partnership between the patient and the healthcare provider is established [4]. Therefore, in person‐centred nursing, the patient–nurse relationship is a core component, with a potential to affect the health outcome of the patient [5]. This relationship affects both the patient and the nurse, encompassing expectations from both parties. Trust is a primary factor, as is the nurse's ability to communicate with the patient. Other influencing components are the attitudes and behaviour of the nurse and their professional knowledge and skills, where not only clinical competence is relevant, but also the ability to support the patient [6]. Similarly, in PCC, the physical care as well as emotional support of the patient are addressed [3]. Lastly, there are contextual and environmental aspects that influence the intricate relationship between the patient and the nurse, which include the social and physical environment of care delivery [6].

There is a widespread use of digital technology within nursing, for example, in the domain of telehealth [7]. One area of use within telehealth is remote patient monitoring (RPM) [8], which can be used with the purpose of surveilling the health of the patient outside the clinical setting [9]. Remote monitoring consists of a structured monitoring of pre‐defined parameters. These parameters include data related to symptoms, behaviour or events, or biological data (measured non‐invasively or invasively). At the other end of data collection, a human recipient is presumed [10]. The COVID‐19 pandemic has expedited the use of RPM, and RPM is forecasted to expand substantially over the next years [11].

RPM should be seen as a complement to, rather than a replacement of, face‐to‐face visits [12], and it can have the ability to strengthen the therapeutic relationship [13]. In a systematic review, Radhakrishnan et al. [14] identified facilitators and barriers for maintaining tele‐homecare programmes, including RPM. The results showed that patient‐tailored adaptations, as well as the relationship between the patient and the nurse, in terms of communication and collaboration, influenced the sustainability of the programmes. This aligns with research showing personalisation by communication as a promoter for patient uptake on digital tools [15]. Similarly, Greenhalgh et al. [10] demonstrated patient and healthcare staff's preferences for in‐person meetings, as well as staff's ideas of compromised relationships as barriers to telehealth uptake within heart failure care. A significant issue is to prevent the physically distant patient from becoming de‐personalised and merely a producer of medical information [16], a concern that has been raised by patients in remote monitoring [17]. This can be avoided by preserving the interpersonal relationship in the distant care situation [16]. In fact, the patients' willingness to use RPM is conditioned and reliant on the relationship with healthcare [18], including the importance of not de‐humanising the patient in the process [13]. However, in addition to patients not wanting to lose interpersonal contact, RPM has been experienced as reassuring and empowering [17]. Recent research on RPM has shown it influences patient adherence and safety, but there is no clear picture of how it affects patients' quality of life [19]. It is known that the care environment and how the care is delivered impacts the relationship between the patient and the nurse [6], but in the case of remote monitoring, the patient is not physically in the ward or clinic, nor has the healthcare provider physically at home. Furthermore, there is a considerable difference between gaining information by remote visual communication with a patient, seeing a person's face and taking in the surrounding environment, and remote monitoring solely through receiving pre‐decided health measurements, which misses this other kind of information [10]. The latter can be assumed to reduce the opportunity for interpersonal interaction. Research shows that visual‐based telehealth promotes the interpersonal connection between the patient and the caregiver and is preferred by the patients [20]. Nonetheless, remote contact cannot offer the caring touch [20], an essential element of nursing [21]. This is important to keep in mind in an increasingly technology‐driven surrounding where the ability to meet the fundamental care needs of the patient must be guarded [1]. To the best of the authors knowledge, there is no recent review that broadly examines how adult patients perceive the patient–nurse relationship during remote monitoring of their health data outside the clinical setting, without focusing on specific diagnoses or conditions.

## 2 Aim

*Population*: Adult patients being remotely monitored by nurses through digital device(s) registering health data.

*Concept*: Patients' experiences/attitudes/perspectives/perceptions of the interpersonal relationship with the nurse in the given care situation.

*Context*: Out‐of‐clinic setting.

This scoping review aimed to explore what has been published in peer‐reviewed journals on patients' experiences of the interpersonal relationship between the patient and the nurse when the patient's health data are remotely monitored in an out‐of‐clinic setting.

## 3 Method

In order to meet the aim, a scoping review was conducted, basing the work on the method of the Joanna Briggs Institute, JBI [22]. The JBI method for conducting a scoping review originates from the framework developed by Arksey and O'Malley [23]. The PRISMA‐ScR checklist [24] was used in the reporting process (File S3). The study was registered at Open Science Framework (10.17605/OSF.IO/T75DN).

### 3.1 Search Procedure

Together with a librarian, a literature search was conducted on September 12th, 2024, in PubMed, CINAHL, Scopus and Web of Science Core Collection. The search aimed for peer‐reviewed, empirical studies published in English between 2014 and September 12th, 2024. The period was chosen with consideration for the acceleration of remote monitoring devices in recent years [11]. The language criterium was due to feasibility and the widespread establishment of literature published in English. The reason for excluding grey literature, conference proceedings and study protocols was that the present study had scientific publications in terms of research articles with existing results in its scope. The search string for each database consisted of three blocks covering Type of technology, Area of use and Patients' experiences/relations. The search process had an iterative nature, which can be the case for scoping reviews [22]. The search string for each database is presented in File S1.

The search strategy was developed from the pre‐defined Population‐Concept‐Context, as recommended by JBI [22].

### 3.2 Selection of Sources of Evidence

Initially, a pilot screening of 33 abstracts from the full database search was conducted, with high interrater reliability. Then all titles and abstracts of the full database search were divided amongst the six authors, working in pairs. Each pair was assigned year‐wise abstracts to screen in a blind mode. Each pair consisted of at least one registered nurse, and the screening was initiated with a screening session with one member from each team. The screening tool Rayyan was used for the screening procedure. Disparities were discussed and solved within each pair. The abstracts included after screening were read in full text to select the studies that qualified to meet the aim. The full texts were read pairwise and independently, and the final studies were selected. Predefined exclusion labels were used when excluding full‐text articles, and disparities regarding inclusion or exclusion were discussed between the authors.

### 3.3 Inclusion and Exclusion Criteria

An inclusion criterion was ‘Nurses (midwives included) exclusively or as part of a multi‐professional team’. That means the articles could cover nurses that were part of a monitoring multiprofessional team. In these cases, where the patient referred to monitoring staff as a group, the results have been accounted as validly associated with the patient–nurse relationship. Regardless of team formation, the nurses and midwives are called ‘nurses’, ‘healthcare provider’ or ‘staff’. See Table 1 for inclusion and exclusion criteria.

**TABLE 1:** Inclusion and exclusion criteria.

	Inclusion criteria	Exclusion criteria
Population	Patients 18 years and over	Children or adolescents (< 18 years)
Concept	Patients' experiences/attitudes/perspectives/perceptions of the patient–nurse relationship	Studies focusing solely on health care professionals' or informal carers' experiences of the patient–nurse relationship, Studies focusing solely on relationships between patients and other professions than nurses, Patients' experiences/attitudes/perspectives/perceptions of the technical function of the device, Patients' perceptions of RPM from a theoretical basis
Context	Out‐of‐clinic setting, Health data collected through remote patient monitoring, by nurses (midwives included) exclusively or as part of a multiprofessional team	Clinical settings (including nursing homes and assisted living facilities), Primary monitoring through video meetings, live chats and telephone calls, Monitoring of medical adherence, Video surveillance, Alarms for personal security: mat alarms, personal alarms, movement detectors etcetera
Type of sources of evidence	Original peer‐reviewed studies in English, published year 2014–2024	Non‐English literature, reviews, research protocols, conference proceedings and grey literature, Studies where abstract does not indicate that the patient experience of the patient–nurse relationship will be an outcome

In contrast to systematic reviews, scoping reviews are not obliged to include quality assessments as part of the methodology [23]. In fact, due to the literature mapping objective of the present scoping review, a quality assessment was not necessary for this aim, in alignment with best practice by Peters et al. [25].

### 3.4 Data Charting and Data Items

Data charting was mainly based on the JBI methodology [22, 26], through an iterative process. A modified version of the *JBI template source of evidence details, characteristics and results extraction instrument* [22] was used when charting the data. The template contained sections for scoping review details, details of the evidence source and results of the evidence source. The template is presented in File S2. Data charting from all relevant full‐text articles was performed pairwise and independently, as in previous steps. Afterwards, the authors went through the extracted data for verification.

### 3.5 Synthetisation of Results

Details of what data was charted are presented in File S2 and results relevant to the objective of the review are presented in Tables 2, 3, 4. Key findings in relation to the aim of the study were analysed using thematic analysis, based on Braun and Clarke's [27] procedure. Firstly, the key findings as extracted by each pair were read and coded by the first author and then discussed with the last author. Secondly, constructed codes were compared based on their differences and similarities and then re‐arranged into distinct themes. Thirdly, the themes were compared based on their differences and similarities and grouped under one of three clusters. For coding structure, see Table 4.

**TABLE 2:** Main characteristics.

Study	Diagnosis or condition	Type of device	Age	Methods	Sample size intervention	Geographical area
Auton et al. [28]	Heart failure	Smartphone app	Mean age 62	Mixed methods: qualitative free‐text thematic analysis, quantitative analysis of vital signs	(*n* = 79*) F (28) M (51)	United Kingdom
Bendix et al. [29]	High‐risk pregnancy	Tablet with technical and clinical equipment	24–40 years (+2 unknown)	Qualitative: interview	(*n* = 15*) F (15)	Denmark
Crafoord et al. [30]	Breast cancer (BC), prostate cancer (PC)	App on smartphone or tablet	BC: 27–73 years, mean age 47 PC: 44–81 years, mean age 72	Mixed method: Questionnaire, medical record, logged data, interview	(*n* = 149) F (74) M (75)	Sweden
Fairbrother et al. [31]	Chronic heart failure	Webpage linked to devices	50–80 years, mean age 75	Qualitative: interview	(*n* = 18) F (7) M (11)	United Kingdom
Goransson et al. [32]	Elderly receiving home care	App on smartphone or tablet	70–101 years, mean age 86	Mixed methods: questionnaire and interview	(*n* = 17*), F (11) M (6)	Sweden
Gordon et al. [33]	Heart failure, hypertension and/or diabetes	App connected to device	44–91 years mean age 73.8	Mixed method: questionnaire, interview, server data, chart data	(*n* = 17*) F (9) M (8)	Canada
Hallberg et al. [34]	Hypertension	Mobile phone platform, web‐based feedback system, monitoring device	43–72	Qualitative: Interview	(*n* = 20) F (11) M (9)	Sweden
Hayden et al. [35]	COVID‐19	Electronic health record mobile or web app (or daily phone call from nurse)	Mean age 57	Quantitative: questionnaire via telephone	(*n* = 180*) F (101) M (79)	USA
Helleman et al. [36]	ALS	App on smartphone, table or computer	Mean age 61	Mixed methods: questionnaire and interview	(*n* = 50) F (18) M (32)	Netherlands
Hyams et al. [37]	Pregnancy diabetes	Smartphone app	Mean age cohort 1: 33.5, cohort 2: 32.4	Quantitative: questionnaire and electronic note	(*n* = 98) F (98)	United Kingdom
Jones et al. [38]	Pregnancy related hypertension	Blood pressure cuff and weight scale	18–50 years	Mixed methods: questionnaire and interview	(*n* = 11) F (11)	USA
Lie et al. [39]	Type II diabetes	Web portal	39–64 years, mean age 51	Qualitative: interview	(*n* = 10) F (6) M (4)	Norway
Liljeroos et al. [40]	Heart failure	Implantable cardioverter‐defibrillator	Mean age 69.9	Mixed methods: questionnaire	(*n* = 175) F (37) M (138)	Sweden
Maguire et al. [41]	Malignant pleural mesothelioma	Smartphone	55–84 years (+1 unknown), mean age 71.6	Mixed method: questionnaire, interview and focus group	(*n* = 18) F (5) M (13)	United Kingdom
McGloin et al. [42]	Type II diabetes	A hub/device to collect health data	37–80 years	Mixed method: focus group and questionnaire	(*n* = 39) > 50% male.	Ireland
Mooney et al. [43]	Cancer	Telephone connected to automated system	Mean age 54.77	Mixed method: questionnaire and interview, data on calls and attrition	(*n* = 180) F (135) M (45)	USA
Morichelli et al. [44]	Implanted ICD	Patient monitor connected to mobile or analogue telephone connection	Mean age 71	Quantitative: questionnaire	(*n* = 163) F (16) M (147)	Italy
Nancarrow et al. [45]	Chronic disease(s)	Tablet connected to monitoring devices	48–98 years, mean age 74.8	Mixed method: questionnaire, interview and focus group	(*n* = 200) F (117) M (83)	Australia
Oelschlagel et al. [46]	Incurable cancer (palliative care)	Tablet and measuring devices	30–94 years, mean age 66	Qualitative: interview	(*n* = 11) F (5) M (6)	Norway
Payakachat et al. [47]	Pre‐eclampsia	Devices for monitoring	No info on age	Qualitative: interview	(*n* = 37) F (37)	USA
Pekmezaris et al. [48]	Type II diabetes	Tablet	No info on age	Qualitative: focus group and interview	(*n* = 12)	USA
Piras and Miele [49]	Type I diabetes	Smartphone app	No info on age	Qualitative: analysis of text message and interview	(*n* = 25) F (25, 10 pregnant)	Italy
Reading et al. [50]	Atrial fibrillation	Device connected to smartphone	Approximately 50–76 years	Qualitative: focus group and interview	(*n* = 7) Approximately 85% male	USA
Sten‐Gahmberg et al. [51]	Chronic disease(s)	Tablet and home telemonitoring device	Most patients > 65 years.	Qualitative: interview	(*n* = 48) Approximately 67% male.	Norway
Strandberg et al. [52]	Heart failure or hypertension	App on smartphone or tablet	50–88 years, mean age 72.5	Qualitative: interview	(*n* = 20), F (9) M (11)	Sweden
Teo et al. [53]	Hypertension or hypertension with hyperlipidemia	Bluetooth‐enabled monitor and mobile data gateway device	Mean age 56.3	Quantitative: questionnaire and registries.	(*n* = 119) F (51) M (68)	Singapore
Teo et al. [54]	Hypertension or hypertension with hyperlipidemia	Bluetooth‐enabled monitor and mobile data gateway device	35–73 years	Qualitative: interview	(*n* = 13) F (5) M (8)	Singapore
Van den Heuvel et al. [55]	High risk pregnancies (PPROM, FGR or preeclampsia)	Wireless device	> 18 years, Mean age 32.1	Qualitative: focus group	(*n* = 11) F (11)	Netherlands
Van Grootven et al. [56]	COVID‐19	Smartphone or tablet	25–84 years	Qualitative: interview	(*n* = 17) ‘Equal’ gender distribution	Belgium
Wathne et al. [57]	Heart failure or colorectal cancer	iPad with app linked to monitoring devices	48–85 years	Qualitative: observation and interview	(*n* = 27) F (8) M (19)	Norway
Wildevuur et al. [58]	Type I diabetes	Wearable device	No info on age	Qualitative: interview	(*n* = 6)	Netherlands

* Not participants enrolled, but those who completed study or participated in researcher follow‐up.

## 4 Results

### 4.1 Search Results

**FIGURE 1:**
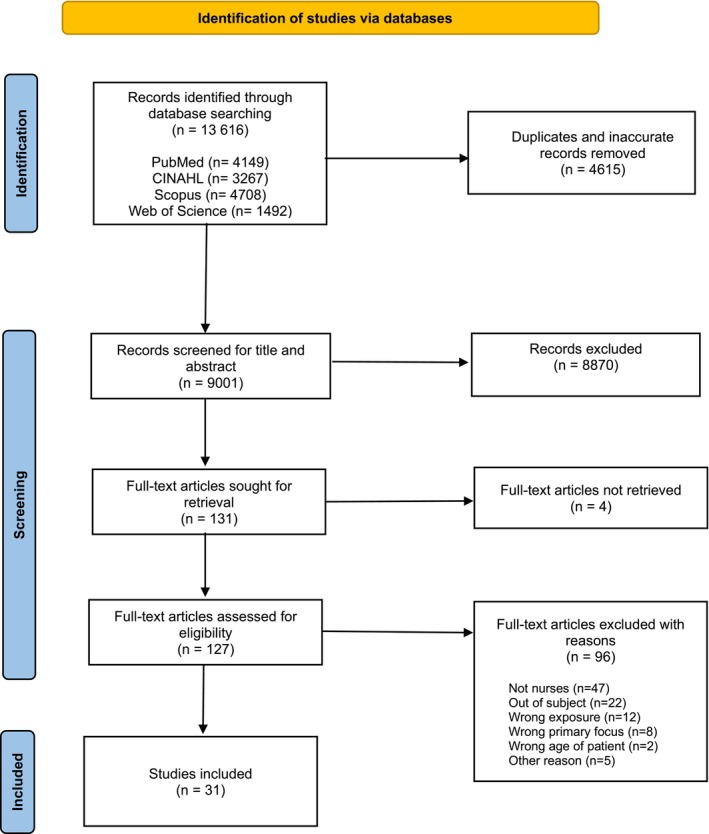
PRISMA flowchart. Modified chart from Page et al. [59] version.

The search in the four databases yielded a total of 13,616 records. After duplicates and obviously faulty results were removed, 9001 titles and abstracts were screened for eligibility and non‐eligible records were removed. This resulted in full‐text screening of 127 articles. Finally, 31 articles were selected for inclusion. See Figure 1 for more information on the screening and selection process.

### 4.2 Characteristics and Results of Selected Studies

**FIGURE 2:**
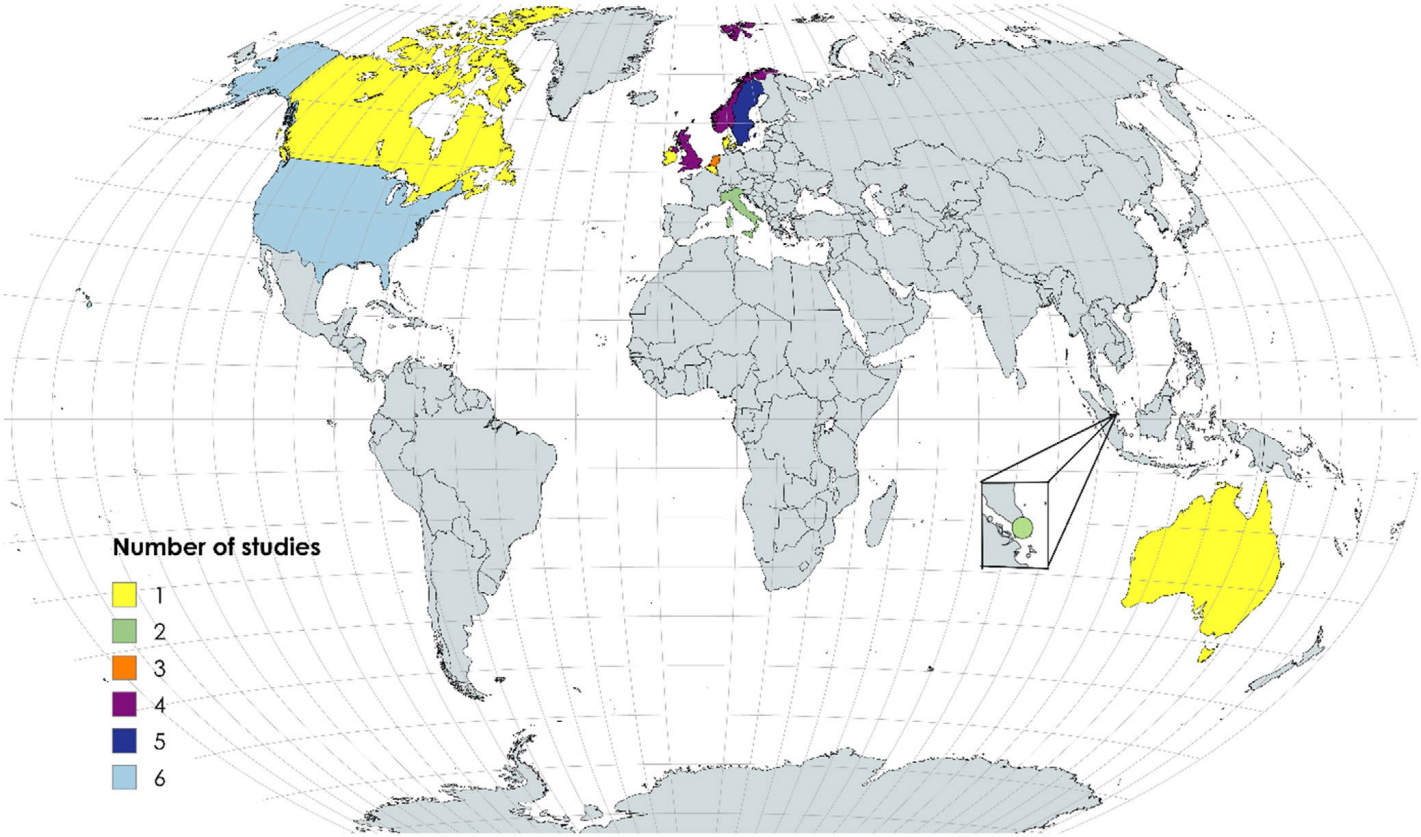
Country of study origin. For one article [44], it is not clear where the study was conducted; therefore, we have categorised this article based on the hospital with which all the authors are affiliated. World map made with MapChart.

As seen in Figure 2, there are twelve countries, represented by four continents in the results of the present review. Scandinavian origin of studies is common, with one third of the selected works originating from these countries.

There is a wide variety of diagnoses or conditions amongst the patients monitored in the studies. Amongst the more common ones are *heart failure* [28, 31, 33, 40, 52, 57], *pregnancies* [29, 37, 38, 47, 55], *diabetes* [33, 39, 42, 48, 49, 58], and *cancer* [30, 43, 46, 57].

As seen in Table 2, the type of device used in the studies varied greatly, with a tablet or mobile phone being used in a majority of the cases [28, 29, 30, 32, 34, 36, 37, 41, 44, 45, 46, 48, 49, 50, 51, 52, 56, 57].

The youngest patients in the review were 18 years of age (see, for example, Jones et al. [38]) and the oldest 101 years of age [32].

Qualitative methods were used exclusively in 16 of the studies, quantitative methods exclusively in four of the studies, and mixed methods in 11. The most common data collection method was interviews (*n* = 24), followed by questionnaires (*n* = 14). The sample size (of the intervention group) varied between six [58] and 200 [45].

As can be seen in Table 3, besides the RPM, most of the articles gave accounts of various ways of contact with patients. In four articles it was not mentioned whether such contacts were used or how such contact was taken [34, 36, 40, 50].

**TABLE 3:** Characteristics of intervention.

Characteristics		*n*	Articles
Additional use of video/chat/text messages/phone			
	Yes	27	Auton et al. [28], Bendix et al. [29], Crafoord et al. [30], Fairbrother et al. [31], Goransson et al. [32], Gordon et al. [33], Hayden et al. [35], Hyams et al. [37], Jones et al. [38], Lie et al. [39], Maguire et al. [41], McGloin [42], Mooney et al. [43], Morichelli et al. [44], Nancarrow et al. [45], Oelschlagel et al. [46], Payakachat et al. [47], Pekmezaris [48], Piras and Miele [49], Sten‐Gahmberg et al. [51], Strandberg et al. [52], Teo et al. [53], Teo et al. [54], van den Heuvel et al. [55], Van Grootven et al. [56], Wathne et al. [57], Wildevuur [58]
	Unknown	4	Hallberg et al. [34], Helleman et al. [36], Liljeroos et al. [40], Reading et al. [50]
Duration of intervention			
	< 1 month	4	Jones et al. [38], van den Heuvel et al. [55], Van Grootven et al. [56], Payakachat et al. [47]
	1–3 months	14	Auton et al. [28], Crafoord et al. [30], Fairbrother et al. [31], Goransson et al. [32], Hallberg et al. [34], Hayden et al. [35], Maguire et al. [41], McGloin [42], Oelschlagel et al. [46], Pekmezaris [48], Piras and Miele [49], Sten‐Gahmberg et al. [51], Wathne et al. [57], Wildevuur [58]
	4–6 months	6	Gordon et al. [33], Mooney et al. [43], Reading et al. [50], Strandberg et al. [52], Teo et al. [53], Teo et al. [54]
	7–9 months	0	
	10–12 months	3	Hyams et al. [37], Liljeroos et al. [40], Nancarrow et al. [45]
	> 12 months	1	Morichelli et al. [44]
	Not specified or ongoing at time of reporting	3	Bendix et al. [29], Helleman et al. [36], Lie et al. [39]
Comparison group			
	Yes	8	Hyams et al. [37], Mooney et al. [43], Payakachat et al. [47], Pekmezaris [48], Reading et al. [50], Sten‐Gahmberg et al. [51], Teo et al. [53], Van Grootven et al. [56]
	No	23	Auton et al. [28], Bendix et al. [29], Crafoord et al. [30], Fairbrother et al. [31], Goransson et al. [32], Gordon et al. [33], Hallberg et al. [34], Hayden et al. [35], Helleman et al. [36], Jones et al. [38], Lie et al. [39], Liljeroos et al. [40], Maguire et al. [41], McGloin [42], Morichelli et al. [44], Nancarrow et al. [45], Oelschlagel et al. [46], Piras and Miele [49], Strandberg et al. [52], Teo et al. [54], Van Grootven et al. [56], Wathne et al. [57], Wildevuur [58]
Monitoring professions			
	Nurses only	19	Auton et al. [28], Bendix et al. [29], Crafoord et al. [30], Goransson et al. [32], Gordon et al. [33], Helleman et al. [36], Jones et al. [38], Lie et al. [39], Liljeroos et al. [40], Maguire et al. [41], McGloin et al. [42], Mooney et al. [43], Morichelli et al. [44], Nancarrow et al. [45], Payakachat et al. [47], Pekmezaris et al. [48], Sten‐Gahmberg et al. [51], Strandberg et al. [52], Wathne et al. [57]
	Multiprofessional team	12	Hallberg et al. [34], Hayden et al. [35], Hyams et al. [37], Oelschlagel et al. [46], Fairbrother et al. [31], Piras and Miele [49], Reading et al. [50], Teo et al. [53], Teo et al. [54], van der Heuvel et al. [55], Van Grootven et al. [56], Wildevuur et al. [58]

**TABLE 4:** Key findings.

Cluster	Theme	References
Relational aspects of patient–nurse communication and interaction	Availability, regularity and dependency	Auton et al. [28], Bendix et al. [29], Crafoord et al. [30], Fairbrother et al. [31], Goransson et al. [32], Gordon et al. [33], Hallberg et al. [34], Helleman et al. [36], Hyams et al. [37], Lie et al. [39], Liljeroos et al. [40], McGloin et al. [42], Mooney et al. [43], Payakachat et al. [47], Piras and Miele [49], Teo et al. [54], van den Heuvel et al. [55], Wathne et al. [57], Wildevuur et al. [58]
	Beneficial communication	Bendix et al. [29], Hayden et al. [35], Lie et al. [39], Payakachat et al. [47], Pekmezaris et al. [48], Piras and Miele [49], Sten‐Gahmberg et al. [51], Van Grootven et al. [56]
	Nurse's active engagement	Mooney et al. [43], Piras and Miele [49], Reading et al. [50], Teo et al. [54], van den Heuvel et al. [55], Van Grootven et al. [56]
	Face‐to‐face interaction	Auton et al. [28], Bendix et al. [29], Lie et al. [39], Liljeroos et al. [40]
Emotional aspects of the patient–nurse relationship	General relationship satisfaction	Gordon et al. [33], Hyams et al. [37], Lie et al. [39], Mooney et al. [43], Morichelli et al. [44], Piras and Miele [49], Sten‐Gahmberg et al. [51], Strandberg et al. [52]
	Protection	Auton et al. [28], Bendix et al. [29], Crafoord et al. [30], Fairbrother et al. [31], Goransson et al. [32], Gordon et al. [33], Hallberg et al. [34], Hayden et al. [35], Hyams et al. [37], Jones et al. [38], Liljeroos et al. [40], Maguire et al. [41], McGloin et al. [42], Mooney et al. [43], Nancarrow et al. [45], Oelschlagel et al. [46], Pekmezaris et al. [48], Piras and Miele [49], Sten‐Gahmberg et al. [51], Strandberg et al. [52], Teo [53], Teo et al. [54], van den Heuvel et al. [55], Van Grootven et al. [56], Wathne et al. [57]
	Closeness	Bendix et al. [29], Crafoord et al. [30], Fairbrother et al. [31], Hayden et al. [35], Lie et al. [39], Liljeroos et al. [40], Maguire et al. [41], Mooney et al. [43], Oelschlagel et al. [46], Piras and Miele [49], Van Grootven et al. [56], Wathne et al. [57]
	Not wanting to be a burden	Crafoord et al. [30], Liljeroos et al. [40]
	Ending intervention	Crafoord et al. [30], Goransson et al. [32], Gordon et al. [33], Wathne et al. [57]
Patient participation		Bendix et al. [29], Crafoord et al. [30], Gordon et al. [33], Hallberg et al. [34], Hyams et al. [37], McGloin et al. [42], Reading et al. [50], Sten‐Gahmberg et al. [51], Wildevuur et al. [58]

The duration of the RPM intervention varied between a few weeks [38, 47, 55, 56] and more than a year [44]. In three of the articles, the duration of intervention was not specified or still ongoing at the time of the reporting [29, 36, 39].

Eight of the articles included a comparison group alongside the intervention group [37, 43, 47, 48, 50, 51, 53, 56]. Of these, four were randomised control trials [43, 48, 50, 51] in which most patients in the control groups received ‘usual care’.

In approximately two thirds of the studies, nurses were responsible for monitoring the patients; but this did not by default exclude physician involvement in patient treatment. In the remaining studies, nurses were part of a multi‐professional monitoring team.

### 4.3 Synthesis of Results

**FIGURE 3:**
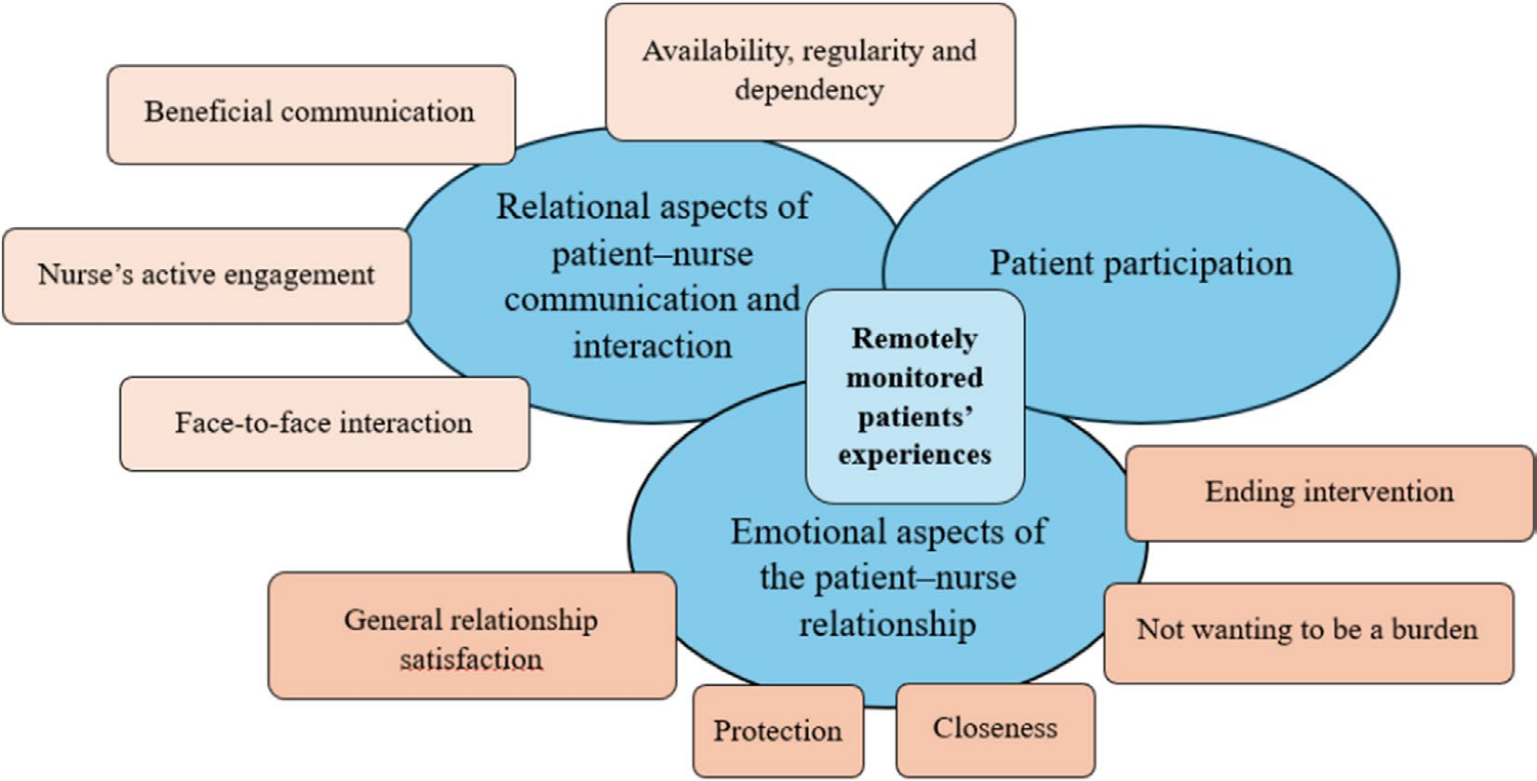
Illustration of clusters and underlying themes in remotely monitored patients' experiences of the interpersonal patient–nurse relationship. The nuances of colours (blue and orange) represent the overlap of clusters and the intra−/interconnection of the themes.

Several of the findings describe a state of the relationship, rather than a change in it. The descriptions are presented in three clusters with enclosed themes below. The clusters and themes are also illustrated in Figure 3.

#### 4.3.1 Cluster 1: Relational Aspects of Patient–Nurse Communication and Interaction

This first cluster includes patients' perceptions of communication or interaction with the nurse in a remote monitoring context.

*Availability, regularity and dependency* include issues of contact frequency, continuity and dependency. Communication increased during the RPM intervention [33, 37, 58]. Many patients in Liljeroos, Thylen and Stromberg [40] valued the reduction of in‐clinic visits to nurses, whereas a minority wanted increased visits. Payakachat et al. [47] found that there were patients who experienced increased stress due to increased call frequency from the nurses. Patients experienced swift answers during the intervention [33] and that RPM had provided continuity of care [28, 31, 36]. Although, in Bendix, Heinsen & Backhausen [29], some patients described a sense of ‘being on‐hold’ and ‘locked‐up’ (p. 4) when staff did not follow‐up as scheduled. In Teo et al. [54] some patients expressed that absence of feedback led to a lack of confidence in their healthcare provider.

*Beneficial communication* addresses the way the nurse delivered information and interacted with the patient. Communication was perceived as responsive [49], patient‐centred [35], and easy and purposeful [47]. RPM could facilitate open communication [39], contributing to social interaction [56]. Additionally, communication was experienced as effective and clear [51], which was valued by the patients [29]. In Pekmezaris et al. [48] patients expressed the importance of culturally adapted communication.

*Nurse's active engagement*. An engaged healthcare provider was believed to contribute to patient engagement [50] and positive experiences of care [56] as well as help the patient to navigate [54]. Moreover, staff were described as attentive [49] and showing concern and empathy [43, 55].

In *Face‐to‐face interaction*, ‘real‐life’ meetings are addressed. Some patients appreciated face‐to‐face contact with the nurses [40] since it made them feel that they were the focus of the meeting [29]. Lie et al. [39] saw that in addition to monitoring, in‐person meetings were expressed as essential for establishing closeness and trust.

#### 4.3.2 Cluster 2: Emotional Aspects of the Patient–Nurse Relationship

The second cluster focuses on patients' emotions when being monitored by the nurse and includes experiences of general relationship satisfaction. In addition, patients' experiences of protection, closeness, not wanting to disturb, and concerns regarding life post‐intervention in a remote monitoring context are addressed.

In *General Relationship Satisfaction* many of the articles, patients reported satisfying relationships with healthcare providers during RPM interventions [39, 43, 44, 49], some even reported enhanced satisfaction with such relationships [33, 37, 51, 52].

In *Protection*, feelings of safety and security, trust, being looked after and supported are addressed as are feelings of unsafety, uncertainty and exposedness. Patients experienced a sense of safety and security during RPM [28, 29, 30, 31, 32, 34, 40, 42, 52, 54, 56], for example that ‘someone is keeping an eye on them’ ([45], p. 644). Patients felt a sense of reassurance during RPM intervention [33, 40, 41, 42, 45, 55, 57]. Moreover, they experienced that they were supported by the healthcare providers [35, 37, 42, 43, 51, 52, 56]. Similarly, in Piras and Miele [49], patients felt that healthcare providers were encouraging and emotionally supportive, they felt cared for and accompanied in their illness, which resulted in a trusting relationship. Participants in Gordon et al. [33] and Teo et al. [53] expressed trust in the providers competence, for example, that they had a responsibility to act when needed [38]. Patients in van der Heuvel et al. [55] stated that they felt that staff were ‘very competent’ (p. 6). However, in Strandberg et al. [52] some patients felt unsafe because of their lack of knowledge in managing monitoring, moreover, a lack of guidance led to uncertainty amongst some participants. Patients could be hesitant in contacting healthcare providers for psycho‐social issues, since it made them feel uncomfortable and exposed [46]. Notably, in Mooney et al. [43] there was a significant increase in mental well‐being for male participants exposed to the RPM intervention, compared to males in the control group. This difference was not found in female participants.

*Closeness* addresses the perceived closeness and being seen, as well as the desire to know the healthcare provider, but also the experience of loneliness. Some patients expressed a sense of closeness to the staff during the RPM intervention [29, 49]. This was similarly described in terms of feeling connected to their healthcare providers [41, 43, 49] – which patients in Oelschlagel et al. [46] considered to be essential to managing their illness. Patients highlighted the importance of knowing one's healthcare provider [31, 57]. They felt seen and acknowledged [30, 35, 49] and even less alone [56]. In contrast, in Liljeroos, Thylen and Stromberg [40], some patients expressed feelings of loneliness during RPM.

In *Not wanting to be a burden* patients hesitated contact with the healthcare providers. Some patients expressed a fear to bother or disturb the nurses if they had to contact them, or even when they had measured deviating values [30, 40].

In *Ending intervention*, patients' feelings of finalising the intervention are addressed. In four studies, patients expressed that they were pleased with RPM and that they wanted to continue after the intervention [30, 32]; some even expressed a worry about what would happen when the intervention ended, since they appreciated the nurse looking out for them during RPM [33, 57].

#### 4.3.3 Cluster 3: Patient Participation

The final cluster has no themes and encompasses patients' perceptions of teamwork and their own roles, including responsibilities, within a remote monitoring context. RPM can lead to a sense of shared and increased responsibility [29, 34, 42], collaboration and involvement in their own care [30, 37, 50, 51] as well as increased compliance [33]. In Wildevuur et al. [58], patients mention that partnership is pivotal. Yet, patients also sensed that RPM could lead to a burden of being in charge as a patient [29].

## 5 Discussion

### 5.1 Distribution of Diagnoses, Sex and Age

The included studies in this review presented various diagnoses and conditions. The most common condition is various cardiovascular diseases. Cardiovascular conditions have been listed as one of the most common conditions for RPM, according to Malasinghe et al. [60]. Although their study is not recent, this trend appears stable. Overall, the present review presents a broad representation of somatic diseases and conditions, which sometimes overlap. Notably, no psychiatric condition is included, although RPM can be used for monitoring mood disorders [61].

The COVID‐19 pandemic resulted in an increased adoption of telehealth [62]. COVID‐19 broke out worldwide during the latter half of the selected search period, and several COVID‐related studies appeared in the search results. In the end, only two of the included studies, Hayden et al. [35] and Van Grootven et al. [56], cover patients with the condition. These patients had a positive experience with communication during RPM and felt supported. It has been shown that patients widely accepted technology‐driven care due to the circumstances of the pandemic, experiencing increased communication and connectedness as a result [62]. Especially in situations of isolation, remote contact can benefit the psychological experience for the patient, as shown by Van Grootven et al. [56].

In studies where detailed information on the sex distribution of the participants is available, the distribution between females and males is even, with a slight overrepresentation of male participants (55%). One included study [43] showed beneficial aspects of RPM with additional nurse support, when it came to the mental health of male patients. Interestingly, Haddad et al. [63] have shown female participants feeling significantly less ready to end RPM, compared to male participants, meanwhileexperiencing staff availability to a lower degree than the males. Additionally, the age spans of the participants in the included studies vary; with some exceptions, the study populations being mainlymiddle‐aged or older. The presence of younger age groups can be partly attributed to studies related to pregnancy. When taking age aspects into consideration, Haddad et al. [63] found significant age differences, with age groups 35 years and above finding the RPM equipment helpful for their home care, compared to the youngest group (18–34). Notably, the eldest age group (≥ 75) expressed to a higher degree an absence of clear explanations from the health care staff regarding when to seek medical attention, compared to younger age groups.

### 5.2 Three Clusters

In the thematised results, amplifiers as well as challenges to the patient–nurse relationship appear when the patient perspective is in focus. In thematic analysis, the themes (in present study named *Clusters*) have a horizontal relationship [27]. In alignment with this, the clusters in present review are to some degree interconnected, for example, communication with the nurse (Cluster 1) affects the emotions and involvement of the patient (Cluster 2 and 3).

#### 5.2.1 Relational Aspects of Patient–Nurse Communication and Interaction

The results show that contact with the nurse during RPM can lead to a higher degree of disease awareness [51]. The participants in the included studies had various frequencies of contact with their healthcare providers during RPM. The importance of being followed up as scheduled was highlighted and not something the patients always could take for granted. Interestingly, frequent contact was not always perceived as positive and could increase feelings of stress for the patient. Similarly, Serrano et al. [9] bring up being constantly reminded of one's disease when participating in RPM as a potential stressor. Others have pointed out that RPM can provide accessibility [64] but also be perceived as overwhelming for the patient and present an additional burden [17] in an already strained situation [65]. Other research, covering chronic and acute conditions, shows that merely a minority (3%) claim difficulties reaching the staff during RPM [63]. How follow‐up routines are designed, implemented and maintained affect not only actual availability, but likely presumptions of availability, and should be considered when delivering RPM.

In Pekmezaris et al. [48] patients appreciated interacting with a nurse speaking their native language. The issue of cultural adaptation, which depends on the patient's cultural background, was not highlighted in the other studies. Furthermore, none of the studies were from Africa or South America, while two represented an Asian country. With one‐third of the studies having Scandinavian origin, this may limit the generalisability to other contexts and cultures.

The results highlight the desire for face‐to‐face contact, which can be viewed as the opposite of the remote monitoring situation. The importance of meeting in real life has been addressed by Walker et al. [17] and Niela‐Vilen et al. [12] where in the latter RPM was emphasised by patients as a complement and not a replacement for traditional care. In contrast, patients in a study by Lundell et al. [64] stated that human contact was essential, albeit not necessarily by interacting face‐to‐face.

When being monitored from a distance, a pivotal matter for patients' experiences is contact with the nurse in the given care situation. This relationship could promote positive experiences and affect the patient's outcome.

#### 5.2.2 Emotional Aspects of the Patient–Nurse Relationship

Feeling supported and secure during RPM is a common perception in the review. Sten‐Gahmberg et al. [51] demonstrate how this support can empower the patient. Unsurprisingly, patients in the review report higher satisfaction scores for interaction with a nurse than with automated coaching support [43], which confirms the constitution of interpersonal relationships as a corner stone of person‐centred nursing [5]. RPM patients reporting a sense of security and safety, and that someone is keeping an eye on them has been demonstrated in previous research [64, 65]. Naturally, this relationship enablement can be due to additional modes of follow‐up (e.g., phone calls) that complement the monitoring device delivering health data, as shown by Chu et al. [65], where participants showed great satisfaction with nurses' phone calls. The presence of additional interaction tools was familiar in the review as well, playing an important role as an enabler of follow‐up. The results showed that RPM sometimes requires a degree of technical skills, which the monitored patient may find challenging, especially when guidance from the healthcare provider is lacking. Interestingly, this mode of care delivery can make the patient feel alone or less alone, as shown in the results. Acknowledging the physical as well as the psychosocial needs of the patient, in an integration of care manner [1], can overcome feelings of loneliness during RPM. Having mental health issues can be perceived as stigmatising [66], which was shown in the present review by Oelschlagel et al. [46], where patients hesitated to talk about these matters with their healthcare provider. This is important for healthcare providers to be aware of and to open up for conversations regarding mental wellbeing, ‘seeing beyond the immediate needs’ ([67], p. 36), rather than merely focusing on the somatic status of their monitored patient. Also, it is of greatest importance that patients do not fear being a burden for the nurse in the monitored situation. In fact, the individual should be involved in, and have power over, their care [4]. This includes effortlessly making contacts when needed. Some patients in the present review mentioned the fear of ending the temporal RPM, since the intervention made them feel safe. It is of ethical concern if patients get used to being monitored, even if only temporarily, and the removal leads to distress or even feelings of being abandoned.

#### 5.2.3 Patient Participation

An informed patient is better equipped to take responsibility for their own health. The results show that RPM has a positive influence on active patient participation and sense of working as part of a team. The contact with the nurse during RPM motivated the patients and contributed to empowerment [42]. Previous research has shown that patients from a low educational background feel more informed about upcoming care by their healthcare provider when enrolled in an RPM programme, compared to patients with a higher educational level [63]. In the present study, data on educational level was not abstracted. Moreover, the results show that RPM can lead to an excessive sense of responsibility for the individual [29], similar to the results found in Walker et al. [17], where expectancies of technology knowledge could lead to an increased burden for the patient. To keep the patient motivated to RPM, it is crucial that the patient is involved at a level that is found manageable and empowering for that person.

### 5.3 RPM In Relation to Person‐Centred Care

This review confirms previous research that RPM can make the patient more disease aware and empowered in managing their condition [9, 12, 17]. By making the patient a personalised and active partner in care, routinisation of care can be avoided and fundamental care needs met [1]. In the present study, the authors used the term ‘patient–nurse’ relationship, despite ‘nurse–patient’ being more commonly established. This choice reflects the emphasis on the patients’ perspective and aligns with PCC emphasising what is important for the unique individual [67]. Nevertheless, the term ‘nurse–patient’ was used in the searches and was considered valid when reviewing the literature. It has been claimed that if the staff has not seen the patient, it can be difficult to get to know them [68]. For patients, this personal knowledge is of importance [64]. This is reflected in the present review, where patients emphasised this matter. Patient knowledge and relationship building is a long‐term process [68]; thus, the nurse must invest time and personal engagement in the patient, whether physically near or at a distance. The human skills of empathy and emotional support cannot be replaced by technology [12], and it should be used without it negatively affecting the interpersonal relationship between the patient and the nurse [21]. This is not just up to the individual nurse but an organisational matter, where decision‐makers must facilitate for care staff to engage in their patients [69].

The review includes all aspects of the PCC core: patient involvement, clinician–patient relationship and context of care delivery [3]. By focusing on the patients' perspective, the voices of the patients are heard when examining the patient–nurse relationship in a distant care setting. Not only is the delivered care gained by a person‐centred approach, so is the research.

### 5.4 Implications

This review shows the patient–nurse relationship to be mainly a positive experience for patients during RPM. The mode of care delivery can empower patients, making them active participants in their own care. On the other hand, RPM can leave the patient in an exposed position, dependent on others, for example, when waiting for the nurse to make contact or when struggling with technical issues. Ethical considerations must be maintained when bringing RPM into the clinical setting [19]. It is important to keep in mind that RPM may not be the optimal solution for all patients [17, 64] and consider the physical and psychological circumstances of the individual when designing new technology [18]. From a sustainability point of view, expedient use of RPM can facilitate access to healthcare, complement it and ideally lead to care equity [64]. Nursing education should raise PCC when teaching about RPM. In workplaces, nurses and other health care professionals should collectively reflect on how PCC can be present in RPM. Conversely, it should be brought to awareness how RPM can benefit PCC [58], to develop its applicability. These matters should be addressed before introducing RPM, but also when implemented and part of standard care. Most importantly, the views of the patients concerned should be considered, ensuring patient involvement. The results of the review are of importance for nurses working with RPM, a nursing approach that is likely to increase in prevalence. The results are most relevant to other health care professionals engaged in RPM as well, and policymakers. Most importantly, the results can support patients' RPM process, making it a safe and health‐gaining experience, with the unique person at the centre of care.

## 6 Limitations

The final search conducted before the study was registered in OSF, which deviates from the JBI recommendations [22]. However, the time from final search to registration was limited, and the chance of important results being missed was considered low. In the database searches, the term *Telemedicine*, instead of *Telehealth*, was chosen. In PubMed, Telemedicine is an established MeSH term. To compare the results that would have been obtained if the subject heading ‘Telehealth’ had been used in CINAHL, a verification search was performed, which led to the conclusion that the original search had covered a substantial portion of the relevant articles.

A challenge during the screening process was determining what kind of distant care in the review would be considered remote monitoring and what would not. The authors made an early decision to exclude video visits, live chats, and safety surveillance as the main mode of monitoring. The aim was to include studies that distinctly had a component of digital data transfer. It turned out interventions often consisted of several components or means of interaction (e.g., data transition, video follow‐up, telephone calls, educational material). Therefore, it is difficult to determine which specific parts of the intervention impact the patient–nurse relationship, or if it is in fact the combination of the different components that determines this.

The decision to only include full texts that explicitly stated that nurses were involved in monitoring may have influenced the outcome, and screened articles where nurses were merely described as, for example, *clinicians* throughout the text might have been missed. The relationship between the patient and the nurse is pivotal for the present review. Still, in the included articles it is not always clear which aspects of relational matters can be attributed to the nurse specifically, if the nurse was part of a multiprofessional monitoring team, as seen in Table 3. Furthermore, the patient's treatment history, their care situation, or their needs prior to the intervention were not investigated. Different diagnoses and, with that, care needs will likely influence one's relationship with the healthcare provider. In some studies, for example, Mooney et al. [43], nurses monitored the patients daily. On the other hand, in Gordon et al. [33] patients uploaded data daily, weekly, or every second week, depending on diagnosis. The frequency of monitoring could potentially impact the relationship. Even so, there was no aim to identify such a pattern within the present study.

Lastly, when the findings were summarised, drop‐out rates or lost to follow‐up within each individual study were generally not accounted for. Also, focus lay on the outcomes of patients exposed to specific interventions, rather than those of a possible control group.

## 7 Conclusions

Being remotely monitored by a nurse makes the patient feel supported and safe, which is positive for their relationship. At the same time, the relationship can be negatively affected due to the vulnerable and exposed position of the patient. When the care is provided from a distance, with the absence of a caring touch, it is of great importance for the nurse to keep a holistic view of the patient, being aware of how the caring actions and attitudes of the nurse affect the patient. Achieving that, RPM can be person‐centred and benefit the patient–nurse relationship as well as the patient's health. Future research should investigate psychiatry patients' perceptions of the patient–nurse relationship during remote monitoring. Psychiatry is a highly relation‐dependent domain, and therefore it is of great importance to investigate this further. Upcoming studies should focus on the cultural aspects of the patient–caregiver relationship when in a remote monitoring context. Additionally, the patient–nurse relationship after the end of RPM would be of relevance to study.

In conclusion, it is not the technological device per se that can be attributed to the positive experiences of RPM, but the active nurse, following up with the patient, showing concern and forming a team with the patient. This manifests RPM as a foremost human‐to‐human activity, with technology playing a facilitating but not leading role.

## Author Contributions

**Anna Granath:** conceptualization, data curation, formal analysis, investigation, methodology, project administration, validation, writing – original draft. **Stine Eileen Torp Løkkeberg:** investigation, methodology, writing – review and editing. **Wivica Kauppi:** conceptualization, investigation, writing – review and editing. **Fredrik Andersen:** conceptualization, investigation, writing – review and editing. **Leif Sandsjö:** conceptualization, investigation, writing – review and editing. **Erik Eriksson:** conceptualization, formal analysis, investigation, methodology, writing – original draft.

## Funding

This research is part of the Swedish Norwegian collaboration project *Kontiki 2* and has received funding from InterReg (nr. 2022–0030) as well as the University of Borås and Østfold University College.

## Conflicts of Interest

The authors declare no conflicts of interest.

## Supporting information


**Data S1:** scs70166‐sup‐0001‐SupinfoS1.docx.

**Data S2:** scs70166‐sup‐0003‐SupinfoS2.docx.

**Data S3:** scs70166‐sup‐0002‐SupinfoS3.docx.

## Data Availability

The data that support the findings of this study are available from the corresponding author upon reasonable request.
