# Supplementary material for: Remotely Monitored Patients' Experiences of the Interpersonal Patient–Nurse Relationship: A Scoping Review
Source: Scand J Caring Sci. 2025 Dec 15;39(4):e70166. doi: 10.1111/scs.70166 (PMC12705915; doi:10.1111/scs.70166)
Supplement: Supplementary file 1 — Data S1: scs70166‐sup‐0001‐SupinfoS1.docx. [file SCS-39-0-s003.docx]

Supplementary file 1. Search strings CINAHL, PubMed, SCOPUS, Web of Science Core Collection.

| **CINAHL via Ebsco Host, search September 12th 2024** | | |
| --- | --- | --- |
|  | Search word | Number of hits |
| Block 1 *Type of technology* | ((MH "Telemedicine+") OR (MH "Smartphone") OR (MH "Mobile Applications") OR (MH "Digital Health+") OR (MH "Wearable Sensors+"))  OR  (TI ( “Welfare technology” OR wearable* OR sensor* OR smartwatch OR “smart watch” OR Smartwear OR “Smart wear” OR mhealth  OR ehealth OR “mobile health” OR “mobile application*” OR smartphone*))  OR  (AB (“Welfare technology” OR wearable* OR sensor* OR smartwatch OR “smart watch” OR Smartwear OR “Smart wear” OR mhealth OR ehealth “mobile health” OR “mobile application*” OR smartphone*) ) | 129, 539 |
| Block 2 *Area of use* | ((MH "Monitoring, Physiologic+") OR ( MH "home health care") OR ( MH “community living” ) OR ( MH “home environment” ) OR (MH "Self-Management") OR (MH "Self Care") OR (MH "Self Administration"))  OR  (TI ( (telemetry OR “self monitoring” OR "Home monitoring" OR "Distance monitoring" OR "Ambulatory Monitoring" OR "Mobile monitoring" OR "Remote monitoring" OR "Remote patient monitoring" OR "portable monitoring" OR "remote surveillance" OR telesurveillance OR telemonitoring OR "outpatient monitoring" OR "monitoring, outpatient" OR "self tracking" OR "health tracking" OR "digital tracking" OR "continuous monitoring” OR “at home” OR “in home” OR “home health*” OR “home care” OR homecare OR "home based" OR “remote care” OR “self care” OR “self management” OR “self administ*”))  OR  (AB (telemetry OR “self monitoring” OR "Home monitoring" OR "Distance monitoring" OR "Ambulatory Monitoring" OR "Mobile monitoring" OR "Remote monitoring" OR "Remote patient monitoring" OR "portable monitoring" OR "remote surveillance" OR telesurveillance OR telemonitoring OR "outpatient monitoring" OR "monitoring, outpatient" OR "self tracking" OR "health tracking" OR "digital tracking" OR "continuous monitoring” OR “at home” OR “in home” OR “home health*” OR “home care” OR homecare OR "home based" OR “remote care” OR “self care” OR “self management” OR “self administ*”)) | 381, 490 |
| Block 3 *Patients*' *experiences/relations* | ((MH "Life Experiences") OR (MH " Patient Attitudes") OR (MH "Patient Compliance") OR (MH "Patient Satisfaction") OR (MH "Patient Preference") OR (MH “Patient participation”) OR (MH "Nurse-Patient Relations") OR (MH "Interpersonal Relations") OR (MH “communication”) OR (MW "Psychosocial Factors"))  OR  (TI (“Patient* perception*” OR “Patient* satisfaction” OR “Patient* experience*” OR “Patient* attitude*” OR “Patient* perspective*” OR "life experience*” OR “lived experience*” OR “experience* of patient*” OR "Patient Relation*" OR "Interpersonal Relation*" OR “patient interaction*” OR “patient communication*” OR “Human interaction*”))  OR  (AB ( “Patient* perception*” OR “Patient* satisfaction” OR “Patient* experience*” OR “Patient* attitude*” OR “Patient* perspective*” OR "life experiences” OR “lived experience*” OR “experience* of patient*” OR "Patient Relation*" OR "Interpersonal Relation*" OR “patient interaction*” OR “patient communication*” OR “Human interaction*”)) | 881,454 |
|  | 1 AND 2 AND 3 | 4290 |
|  | Filter: English, peer reviewed, 2014-2024 | 3267 |

| **PubMed, search September 12th 2024** | | |
| --- | --- | --- |
|  | Search word | Number of hits |
| Block 1 *Type of technology* | (("Telemedicine"[Mesh] OR "Smartphone"[Mesh] OR "Mobile Applications"[Mesh] OR "Digital Health"[Mesh] OR "Wearable Electronic Devices"[Mesh])  OR  ("Welfare technology"[Title/Abstract] OR wearable*[Title/Abstract] OR sensor*[Title/Abstract] OR smartwatch[Title/Abstract] OR "smart watch"[Title/Abstract] OR Smartwear[Title/Abstract] OR "Smart wear"[Title/Abstract] OR mhealth[Title/Abstract] OR ehealth[Title/Abstract] OR "mobile health"[Title/Abstract] OR "mobile application*"[Title/Abstract] OR smartphone*[Title/Abstract])) | 602, 893 |
| Block 2 *Area of use* | (("Monitoring, Physiologic"[Mesh] OR "Home Care Services"[Mesh] OR "Home Environment"[Mesh] OR "Self-Management"[Mesh] OR "Self Care"[Mesh] OR "Self Administration"[Mesh])  OR  ("community living"[Title/Abstract] OR telemetry[Title/Abstract] OR "self monitoring"[Title/Abstract] OR "Home monitoring"[Title/Abstract] OR "Distance monitoring"[Title/Abstract] OR "Ambulatory Monitoring"[Title/Abstract] OR "Mobile monitoring"[Title/Abstract] OR "Remote monitoring"[Title/Abstract] OR "Remote patient monitoring"[Title/Abstract] OR "portable monitoring"[Title/Abstract] OR "remote surveillance"[Title/Abstract] OR telesurveillance[Title/Abstract] OR telemonitoring[Title/Abstract] OR "outpatient monitoring"[Title/Abstract] OR "monitoring, outpatient"[Title/Abstract] OR "self tracking"[Title/Abstract] OR "health tracking"[Title/Abstract] OR "digital tracking"[Title/Abstract] OR "continuous monitoring"[Title/Abstract] OR "at home"[Title/Abstract] OR "in home"[Title/Abstract] OR "home health*"[Title/Abstract] OR "home care"[Title/Abstract] OR homecare[Title/Abstract] OR "home based"[Title/Abstract] OR "remote care"[Title/Abstract] OR "self care"[Title/Abstract] OR "self management"[Title/Abstract] OR "self administ*"[Title/Abstract])) | [458,827](https://pubmed.ncbi.nlm.nih.gov/?term=%28%22Monitoring%2C+Physiologic%22%5BMesh%5D+OR+%22Home+Care+Services%22%5BMesh%5D+OR+%22Home+Environment%22%5BMesh%5D+OR+%22Self-Management%22%5BMesh%5D+OR+%22Self+Care%22%5BMesh%5D+OR+%22Self+Administration%22%5BMesh%5D+AND+%28english%5BFilter%5D%29%29+OR+%28%22community+living%22%5BTitle%2FAbstract%5D+OR+telemetry%5BTitle%2FAbstract%5D+OR+%22self+monitoring%22%5BTitle%2FAbstract%5D+OR+%22Home+monitoring%22%5BTitle%2FAbstract%5D+OR+%22Distance+monitoring%22%5BTitle%2FAbstract%5D+OR+%22Ambulatory+Monitoring%22%5BTitle%2FAbstract%5D+OR+%22Mobile+monitoring%22%5BTitle%2FAbstract%5D+OR+%22Remote+monitoring%22%5BTitle%2FAbstract%5D+OR+%22Remote+patient+monitoring%22%5BTitle%2FAbstract%5D+OR+%22portable+monitoring%22%5BTitle%2FAbstract%5D+OR+%22remote+surveillance%22%5BTitle%2FAbstract%5D+OR+telesurveillance%5BTitle%2FAbstract%5D+OR+telemonitoring%5BTitle%2FAbstract%5D+OR+%22outpatient+monitoring%22%5BTitle%2FAbstract%5D+OR+%22monitoring%2C+outpatient%22%5BTitle%2FAbstract%5D+OR+%22self+tracking%22%5BTitle%2FAbstract%5D+OR+%22health+tracking%22%5BTitle%2FAbstract%5D+OR+%22digital+tracking%22%5BTitle%2FAbstract%5D+OR+%22continuous+monitoring%22%5BTitle%2FAbstract%5D+OR+%22at+home%22%5BTitle%2FAbstract%5D+OR+%22in+home%22%5BTitle%2FAbstract%5D+OR+%22home+health%2A%22%5BTitle%2FAbstract%5D+OR+%22home+care%22%5BTitle%2FAbstract%5D+OR+homecare%5BTitle%2FAbstract%5D+OR+%22home+based%22%5BTitle%2FAbstract%5D+OR+%22remote+care%22%5BTitle%2FAbstract%5D+OR+%22self+care%22%5BTitle%2FAbstract%5D+OR+%22self+management%22%5BTitle%2FAbstract%5D+OR+%22self+administ%2A%22%5BTitle%2FAbstract%5D+AND+%28english%5BFilter%5D%29%29&filter=lang.english&ac=no&sort=relevance) |
| Block 3 *Patients*' *experiences/relations* | (("Life Change Events"[Mesh] OR "Patient Compliance"[Mesh] OR "Patient Satisfaction"[Mesh] OR "Patient Preference"[Mesh] OR "Patient Participation"[Mesh] OR "Nurse-Patient Relations"[Mesh] OR "Interpersonal Relations"[Mesh:NoExp] OR "Communication"[Mesh:NoExp] OR "psychology"[Subheading])  OR  ("Patient* perception*"[Title/Abstract] OR "Patient* satisfaction"[Title/Abstract] OR "Patient* experience*"[Title/Abstract] OR "Patient* attitude*"[Title/Abstract] OR "Patient* perspective*"[Title/Abstract] OR "life experience*"[Title/Abstract] OR "lived experience*"[Title/Abstract] OR "experience* of patient*"[Title/Abstract] OR "Patient Relation*"[Title/Abstract] OR "Interpersonal Relation*"[Title/Abstract] OR "patient interaction*"[Title/Abstract] OR "patient communication*"[Title/Abstract] OR "Human interaction*"[Title/Abstract])) | 1,670, 037 |
|  | 1 AND 2 AND 3 | 5428 |
|  | Filter: English, 2014-2024 | 4149 |

| **SCOPUS search September 12th 2024** | | |
| --- | --- | --- |
|  | Search word | Number of hits |
| Block 1 *Type of technology* | ( TITLE-ABS-KEY ( ( telemedicine OR smartphone* OR "Mobile Application*" OR "Digital Health" OR wearable* OR "Welfare technology" OR smartwatch OR "smart watch" OR smartwear OR "Smart wear" OR mhealth OR ehealth OR "mobile health" OR "mobile application*" OR smartphone* ) ) ) | 384,708 |
| Block 2 *Area of use* | ( TITLE-ABS-KEY ( ( "physiologic* monitoring" OR "Home Environment" OR "community living" OR telemetry OR "self monitoring" OR "Home monitoring" OR "Distance monitoring" OR "Ambulatory Monitoring" OR "Mobile monitoring" OR "Remote monitoring" OR "Remote patient monitoring" OR "portable monitoring" OR "remote surveillance" OR telesurveillance OR telemonitoring OR "outpatient monitoring" OR "monitoring, outpatient" OR "self tracking" OR "health tracking" OR "digital tracking" OR "continuous monitoring" OR "at home" OR "in home" OR "home health*" OR "home care" OR homecare OR "home based" OR "remote care" OR "self care" OR "self management" OR "self administ*" ) ) ) | 651,728 |
| Block 3 *Patients*' *experiences/relations* | ( TITLE-ABS-KEY ( ( "Patient* perception*" OR "Patient* satisfaction" OR "Patient* experience*" OR "Patient* attitude*" OR "Patient* perspective*" OR "life experience*" OR "lived experience*" OR "experience* of patient*" OR "Patient Relation*" OR "Interpersonal Relation*" OR "patient interaction*" OR "patient communication*" OR "Human interaction*" OR "Life Change Events" OR "Patient* Compliance*" OR "Patient* Preference*" OR "Patient* Participation*" OR "Interpersonal Relations" OR "psychosocial factors" ) ) ) | 986,318 |
|  | 1 AND 2 AND 3 | 5752 |
|  | Filter: English, 2014-2024 | 4708 |

| **Web of Science Core Collection search September 12th 2024** | | |
| --- | --- | --- |
|  | Search word | Number of hits |
| Block 1 *Type of technology* | (Telemedicine OR Smartphone* OR “Mobile Application*” OR “Digital Health” OR Wearable* OR "Welfare technology" OR sensor* OR smartwatch OR "smart watch" OR Smartwear OR "Smart wear" OR mhealth OR ehealth OR "mobile health" OR "mobile application*" OR smartphone*) (Topic) | 1,787,172 |
| Block 2 *Area of use* | ("physiologic monitoring" OR “Home Environment” OR "community living" OR telemetry OR "self monitoring" OR "Home monitoring" OR "Distance monitoring" OR "Ambulatory Monitoring" OR "Mobile monitoring" OR "Remote monitoring" OR "Remote patient monitoring" OR "portable monitoring" OR "remote surveillance" OR telesurveillance OR telemonitoring OR "outpatient monitoring" OR "monitoring, outpatient" OR "self tracking" OR "health tracking" OR "digital tracking" OR "continuous monitoring" OR "at home" OR "in home" OR "home health*" OR "home care" OR homecare OR "home based" OR "remote care" OR "self care" OR "self management" OR "self administ*") (Topic) | 378,348 |
| Block 3 *Patients*' *experiences/relations* | ("Patient* perception*" OR "Patient* satisfaction" OR "Patient* experience*" OR "Patient* attitude*" OR "Patient* perspective*" OR "life experience*" OR "lived experience*" OR "experience* of patient*" OR "Patient Relation*" OR "Interpersonal Relation*" OR "patient interaction*" OR "patient communication*" OR "Human interaction*" OR "Life Change Events" OR "Patient* Compliance*" OR "Patient* Preference*" OR "Patient* Participation*" OR "Interpersonal Relations" OR “psychosocial factors”) (Topic) | 304,873 |
|  | 1 AND 2 AND 3 | 1677 |
|  | Filter: English, 2014-2024 | 1492 |
