# Supplementary material for: Remotely Monitored Patients' Experiences of the Interpersonal Patient–Nurse Relationship: A Scoping Review
Source: Scand J Caring Sci. 2025 Dec 15;39(4):e70166. doi: 10.1111/scs.70166 (PMC12705915; doi:10.1111/scs.70166)
Supplement: Supplementary file 2 — Data S2: scs70166‐sup‐0003‐SupinfoS2.docx. [file SCS-39-0-s001.docx]

**Supplementary file 2.** JBI template source of evidence details, characteristics and results extraction instrument^[[1]](#footnote-1)^ (modified version).

| **Scoping review details** | |
| --- | --- |
| Scoping review title | Remotely monitored patients’ experiences of the interpersonal patient–nurse relationship: a scoping review |
| Review objectives | To explore what has been published in peer-reviewed journals on patients’ experiences of the interpersonal relationship between the patient and the nurse, when the patient’s health data are remotely monitored in an out-of-clinic setting. |
| **Inclusion/exclusion criteria** | |
| Population | Adult patients being remotely monitored by nurses through digital device(s) registering health data |
| Concept | Patients’ experiences/attitudes/perspectives/perceptions of the interpersonal relationship with the nurse in the given care situation |
| Context | Out-of-clinic setting |
| **Evidence source Details and Characteristics** | |
| Citation details (author/s year of publication) |  |
| Country of origin (where study was conducted) |  |
| Methodology (study design and data collection) |  |
| Patient population (diagnosis, age, sample size, sex) |  |
| Technology (type of device, additional use of video/chat/text messages/phone, duration of intervention) |  |
| Comparison group (yes/no) |  |
| Monitoring professions (nurses only/multiprofessional team) |  |
| **Details/Results extracted from source of evidence**(in relation to the concept of the scoping review) | |
| Key findings in relation to scoping review question |  |

1. Peters MDJ, Godfrey C, McInerney P, Munn Z, Tricco AC, Khalil, H. Scoping Reviews (2020). Aromataris E, Lockwood C, Porritt K, Pilla B, Jordan Z, editors. JBI Manual for Evidence Synthesis. JBI; 2024. Available from: https://synthesismanual.jbi.global. https://doi.org/10.46658/JBIMES-24-09 [↑](#footnote-ref-1)
